# Greater exposure to PM_2.5_ and PM_10_ was associated with lower corneal nerve measures: the Maastricht study - a cross-sectional study

**DOI:** 10.1186/s12940-024-01110-1

**Published:** 2024-09-04

**Authors:** Sara B. A. Mokhtar, Jessica Viljoen, Carla J. H. van der Kallen, Tos T. J. M. Berendschot, Pieter C. Dagnelie, Jeroen D. Albers, Jens Soeterboek, Fabio Scarpa, Alessia Colonna, Frank C. T. van der Heide, Marleen M. J. van Greevenbroek, Hans Bosm, Abraham A. Kroon, Rudy M. M. A. Nuijts, Marlies Gijs, Jeroen Lakerveld, Rayaz A. Malik, Carroll A. B. Webers, Coen D. A. Stehouwer, Annemarie Koster

**Affiliations:** 1 Department of Internal Medicine, School for Cardiovascular Diseases, Maastricht University Medical Center, Maastricht, the Netherlands; 2 School of Mental Health and Neuroscience, University Eye Clinic Maastricht, Maastricht University Medical Center, Maastricht, the Netherlands; 3 Department of Social Medicine, Maastricht University, Maastricht, The Netherlands; 4 Care and Public Health Research Institute, Maastricht University, Maastricht, The Netherlands; 5 Alzheimer Centrum Limburg, Department of Psychiatry and Neuropsychology, School of Mental Health and Neuroscience, Maastricht University Medical Center+, Maastricht, The Netherlands; 6 Department of Information Engineering, University of Padova, Padua, Italy; 7 Université de Paris, Inserm U1153, Epidemiology of Ageing and Neurodegenerative Diseases, Paris, France; 8 Heart and Vascular Center, Maastricht University Medical Center, Maastricht, the Netherlands; 9 Global Geo Health Data Center, University Medical Center Utrecht & Utrecht University, Utrecht, the Netherlands; 10 Department of Epidemiology and Data Science, Amsterdam UMC, Vrije Universiteit Amsterdam, Amsterdam, The Netherlands; 11 Department of Medicine, Weill Cornell Medicine-Qatar, Qatar Foundation, Education City, Doha, Qatar; 12 Institute of Cardiovascular Science, University of Manchester, Manchester, UK; 13 Department of Chronic Diseases and Metabolism (CHROMETA), KU Louvain, Louvain, Belgium

**Keywords:** Air pollution, Particulate matter, NO_2_, Elemental carbon, Corneal nerves, Neurodegeneration

## Abstract

**Background:**

Epidemiological and toxicological studies indicate that increased exposure to air pollutants can lead to neurodegenerative diseases. To further confirm this relationship, we evaluated the association between exposure to ambient air pollutants and corneal nerve measures as a surrogate for neurodegeneration, using corneal confocal microscopy.

**Methods:**

We used population-based observational cross-sectional data from The Maastricht Study including *N* = *3635* participants (mean age 59.3 years, 51.6% were women, and 19.9% had type 2 diabetes) living in the Maastricht area. Using the Geoscience and hEalth Cohort COnsortium (GECCO) data we linked the yearly average exposure levels of ambient air pollutants at home address-level [particulate matter with diameters of ≤ 2.5 µm (PM2.5), and ≤ 10.0 µm (PM10), nitrogen dioxide (NO2), and elemental carbon (EC)]. We used linear regression analysis to study the associations between Z-score for ambient air pollutants concentrations (PM_2.5_, PM_10_, NO_2_, and EC) and Z-score for individual corneal nerve measures (corneal nerve bifurcation density, corneal nerve density, corneal nerve length, and fractal dimension).

**Results:**

After adjustment for potential confounders (age, sex, level of education, glucose metabolism status, corneal confocal microscopy lag time, inclusion year of participants, smoking status, and alcohol consumption), higher Z-scores for PM_2.5_ and PM_10_ were associated with lower Z-scores for corneal nerve bifurcation density, nerve density, nerve length, and nerve fractal dimension [stβ (95% CI): PM_2.5_ -0.10 (-0.14; -0.05), -0.04 (-0.09; 0.01), -0.11 (-0.16; -0.06), -0.20 (-0.24; -0.15); and PM_10_ -0.08 (-0.13; -0.03), -0.04 (-0.09; 0.01), -0.08 (-0.13; -0.04), -0.17 (-0.21; -0.12)], respectively. No associations were found between NO_2_ and EC and corneal nerve measures.

**Conclusions:**

Our population-based study demonstrated that exposure to higher levels of PM_2.5_ and PM_10_ are associated with higher levels of corneal neurodegeneration, estimated from lower corneal nerve measures. Our results suggest that air pollution may be a determinant for neurodegeneration assessed in the cornea and may impact the ocular surface health as well.

**Supplementary Information:**

The online version contains supplementary material available at 10.1186/s12940-024-01110-1.

## Introduction

Air pollution significantly impacts health, with an estimated seven million annual deaths in 2021, according to the World Health Organization (WHO) [1]. Efforts have been made to improve air quality by imposing stricter guidelines set by the WHO [1].

Air pollution is a complex mixture of gasses, solid particles, and liquid droplets. The pollutants that have the strongest supporting evidence regarding public health issues encompass particulate matter (PM), ozone, nitrogen dioxide (NO_2_), sulfur dioxide, and carbon monoxide [1]. The broad spectrum complications attributed to air pollution extends beyond respiratory issues [2] to ocular health [3] and neurodegeneration [4]. Previous studies investigated the impact of air pollution ambient level exposure on alterations of the ocular surface [5], dry eye syndrome [6], allergic conjunctivitis [7, 8], keratoconus [9] and corneal morphology [3, 10–12]. Moreover, studies have shown that air pollution can cause neuroinflammation and neurodegeneration [13].

Despite the cornea's direct vulnerability to environmental factors, there is currently a notable gap in our understanding of the association between ambient air pollutant exposure and corneal nerve morphology in well-characterized study populations. The integrity of the corneal nerves can serve as a valuable window for observing corneal health [14], and central and peripheral neurodegenerative disorders [15–20]. Studying this association is important because alterations in corneal sensitivity due to corneal neurodegeneration can lead to dry eye, delayed wound healing after injury, infections, and persistent epithelial defects [21] which may result in severe vision loss or even blindness [22]. Moreover, as postulated in the ticking clock hypothesis, the onset and/or progression of early neurodegeneration can be delayed by reducing exposure to potentially modifiable risk factors [23, 24]. To the best of our knowledge, the association between exposure to air pollutants and neurodegeneration assessed in the cornea has not been studied. In the present paper, we focus on the exposure to air pollutants as a determinant of morphological changes of corneal nerve fibers assessed using the in vivo, non-invasive and sensitive ophthalmic technique of corneal confocal microscopy [25].

Using data from The Maastricht Study we examined the associations of exposure to ambient air pollution estimated at home address with corneal nerve fiber measures, as assessed using corneal confocal microscopy.

## Methods

### Study population and design

**Fig. 1:**
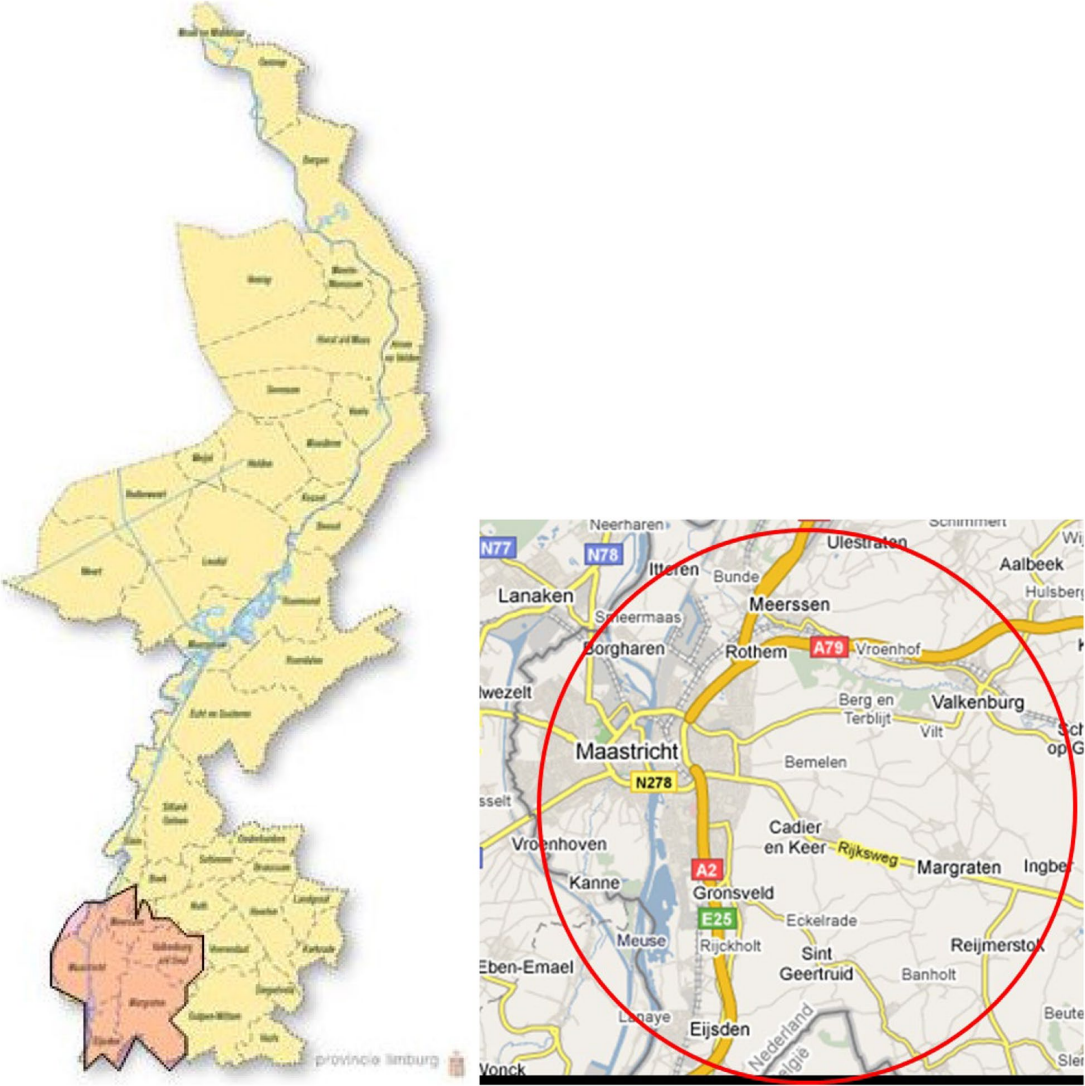
Geographical Area of Participant Recruitment: Maastricht Region

We used data from the Maastricht Study, an observational prospective population-based cohort study enriched with type 2 diabetes individuals, for efficiency reasons. The rationale and methodology have been described previously [26]. In brief, the study focuses on the etiology, pathophysiology, complications and comorbidities of type 2 diabetes mellitus, and is characterized by an extensive phenotyping approach. Eligible for participation were all individuals aged between 40 and 75 years living in the southern part of the Netherlands. Participants were recruited from the Maastricht area, as shown in Fig. 1, through mass media campaigns and from the municipal registries and the regional Diabetes Patient Registry via mailings. Recruitment was stratified according to known type 2 diabetes status [26]. We had cross-sectional data for N = 9187 participants who were included for the baseline survey between November 2010 and October 2020. The examinations for each participant were performed within a time window of 3 months. Estimates of address-level yearly average levels of ambient air pollutants were provided by the Geoscience and Health Cohort Consortium (GECCO) [27]. The study was approved by the institutional medical ethical committee (NL31329.068.10) and the Minister of Health, Welfare and Sports of the Netherlands (permit 131088–105234-PG). All participants gave written informed consent [26].

### Assessment of corneal confocal microscopy measurements

We utilized the Heidelberg retina tomograph III (HRT3) along with the Rostock cornea module (Heidelberg Engineering, Germany) for imaging the corneal nerves [25] of the left eye exclusively, due to logistical considerations. Prior to the assessment, we applied oxybuprocaine hydrochloride 0.4 mg/ml eye drops (Minims; Bausch & Lomb, France) to both eyes to prevent blinking, and applied carbomer 2 mg/g eye gel (Vidsic Bausch & Lomb) to both eyes to ensure optimal contact between the cornea and the applanation cap [20].

We performed large-scale corneal confocal microscopy imaging (1600 × 1600 μm). We employed the U-net-based convolutional neural network for fully automated tracing of the corneal nerves [28] (example can be found in Supplemental Fig. S1). Traced corneal nerve images were reviewed manually to ensure quality criteria were met (example can be found in Supplemental Fig. S2), and the location of the captured images was assessed based on the orientation of the corneal nerves (Supplemental Fig. S3). Additional details regarding this process can be found in the supplementary methods.

Our analysis included the following indices: corneal nerve bifurcation density (number of bifurcation points or branching points per mm2), corneal nerve density (total number of corneal nerve fibers, including main fibers and branches, per mm2; 'main nerve fibers' denoting the largest and most prominent nerve fibers), corneal nerve length (total length of corneal nerve fibers in mm, including both main fibers and branches, per mm2), and corneal nerve fractal dimension (a measure of nerve structure complexity, unit-less). Additional information can be found in the supplementary methods.

### Assessment of air pollution

The assessment of air pollution exposure cannot be captured by a single pollutant due to the diversity of sources and dispersion patterns. Consequently, we selected particulate matter with diameters of ≤ 2.5 µm (PM2.5), and ≤ 10.0 µm (PM10), nitrogen dioxide (NO_2_), and elemental carbon (EC) as key pollutants for evaluation, given their regulatory significance and health implications [29].

The Institute for Public Health and the Environment (RIVM) models and maps the average concentrations of these pollutants every year, using a grid resolution of 25 m. These maps were derived from 1 km resolution nationwide background concentration maps, which were further refined with local traffic data. In essence, the nationwide background maps incorporated dispersion models that included data on emissions from industrial, vehicular, and household sources both within the Netherlands and from neighboring regions, as well as meteorological and chemical information [30]. The local traffic data, sourced from the Dutch Nationaal Samenwerkingsprogramma Luchtkwaliteit, were integrated with the national background maps using two specific models, one for urban roads and another for highways in more open areas [31, 32].

Air pollution concentrations were then computed at 9 million data points across the Netherlands and interpolated to a 25 m resolution raster map using ordinary Kriging, the data are publicly available [33]. The GECCO linked these air pollution concentrations to residential addresses and exported the data into a tabular format. Within GECCO, estimated concentrations of air pollutants were available for the years 2013 to 2020 for PM_2.5_, PM_10_, and EC, or 2014 to 2020 for NO_2_. While the model predictions and absolute concentrations for NO_2_ and PM_2.5_ generally aligned well with measured values [34], the interpretation of quality metrics is complex due to the use of measurements in model calibration. The models for elemental carbon are more recent and less thoroughly calibrated, potentially leading to misclassification of absolute concentrations. The EC maps are based on scaled emissions of PM2.5 and are calibrated using black smoke measurements that are converted to estimated EC values [35]. However, since a potential misclassification is likely consistent across The Maastricht Study population, comparisons of relative differences between groups remain valid.

We linked the estimated annual concentrations of air pollutants to The Maastricht Study participants’ home address at baseline, specifically for the year of the corneal observation. This approach ensures that the exposure assessment is contemporaneous with the corneal nerve assessment. For example: If corneal nerve assessment was conducted in 2015, we linked it to the air pollution levels of 2015.

### Covariates

We determined glucose metabolism status (normal glucose metabolism, prediabetes, type 2 diabetes and other types of diabetes) according to the WHO 2006 criteria, based on a 75-g oral glucose tolerance test and use of glucose-lowering medication [36]. We assessed educational level (low, intermediate, high), occupational level (low, intermediate, high, self-employee, or other), household income level [37], smoking status (never, former, current), alcohol use (none, low, high), history of ocular disorders (corneal diseases or uveitis), and use of contact lenses by questionnaire. The Dutch Healthy Diet index [38] was based on a validated food frequency questionnaire [39]. Urbanity was determined by the average "environmental address density" within a center (summarized to neighborhoods) and classified as (very strong urban (> 2500 addresses per km^2^), strong urban (1500 – 2500 addresses per km^2^), moderately urban (1000 – 1500 addresses per km^2^), limited urban (500 – 1000 addresses per km^2^), non-urban (< 500 addresses per km^2^).

### Statistical analyses

All statistical analyses were conducted using Statistical Package for Social Sciences version 25.0 (IBM SPSS, USA). A significance level of *P* < 0.05 was adopted for all analyses. Population characteristics and corneal nerve measurements were outlined for the entire study cohort and categorized by tertiles of PM_2.5_.

It is important to note that higher levels of air pollution are considered detrimental to health, and similarly, a decrease in corneal nerve measures is indicative of adverse outcomes.

### Main analyses

We employed multivariable linear regression analyses to investigate the associations of Z-scores for PM_2.5_, PM_10_, NO_2_, and EC with Z-scores for corneal nerve bifurcation density, corneal nerve density, corneal nerve length, and fractal dimension.

Corneal confocal microscopy assessments were conducted from April 2013 to October 2020. Previous participants of the Maastricht Study who had been involved before the commencement of corneal confocal microscopy (i.e., prior to April 2013) were re-invited for corneal confocal microscopy examinations (n = 950; 26.1%). Among these participants, there was a median interval of 5.2 years, referred to as the 'visit interval' or 'lag time', between corneal confocal microscopy assessments and the initial baseline covariate measurements (such as glucose metabolism status). We incorporated adjustments for this lag time in our analysis.

We implemented various levels of adjustments in our analysis. Model 1 presents unadjusted results. Model 2 was adjusted for age, sex, level of education, glucose metabolism status, corneal confocal microscopy lag time (the interval between baseline measurements and corneal confocal microscopy scans), and inclusion year (presented as a categorical variable) of participants to control for potential period effects. Additionally, Model 3 included adjustments for smoking status and alcohol consumption. These variables were selected as they are significant potential confounders. All associations are presented as standardized regression coefficients along with their corresponding 95% confidence intervals (CI).

### Additional analyses

To ensure the robustness of our findings, we performed several additional analyses. Firstly, we examined interaction effects with sex and glucose metabolism status by incorporating interaction terms into Model 3. Secondly, we repeated the analyses with additional adjustments for lifestyle factors (dietary intake excluding alcohol and physical activity), ocular variables (corneal diseases, uveitis), use of glasses or contact lenses, and urbanicity. These adjustments were not included in the primary analyses due to missing data for a substantial number of participants. Thirdly, we conducted additional analyses where adjustments were made for the location of captured corneal nerve images (encoded as dummy variables for center, semi-center, and inferior whorl), and the orientation of the corneal nerve fibers (treated as a continuous variable). This was done because the characteristics of corneal nerves vary based on the location of the captured image (e.g., corneal nerve density differs between the inferior whorl, center, and semi-center). Finally, we substituted level of education with either occupational status or income equivalent.

## Results

**Fig. 2:**
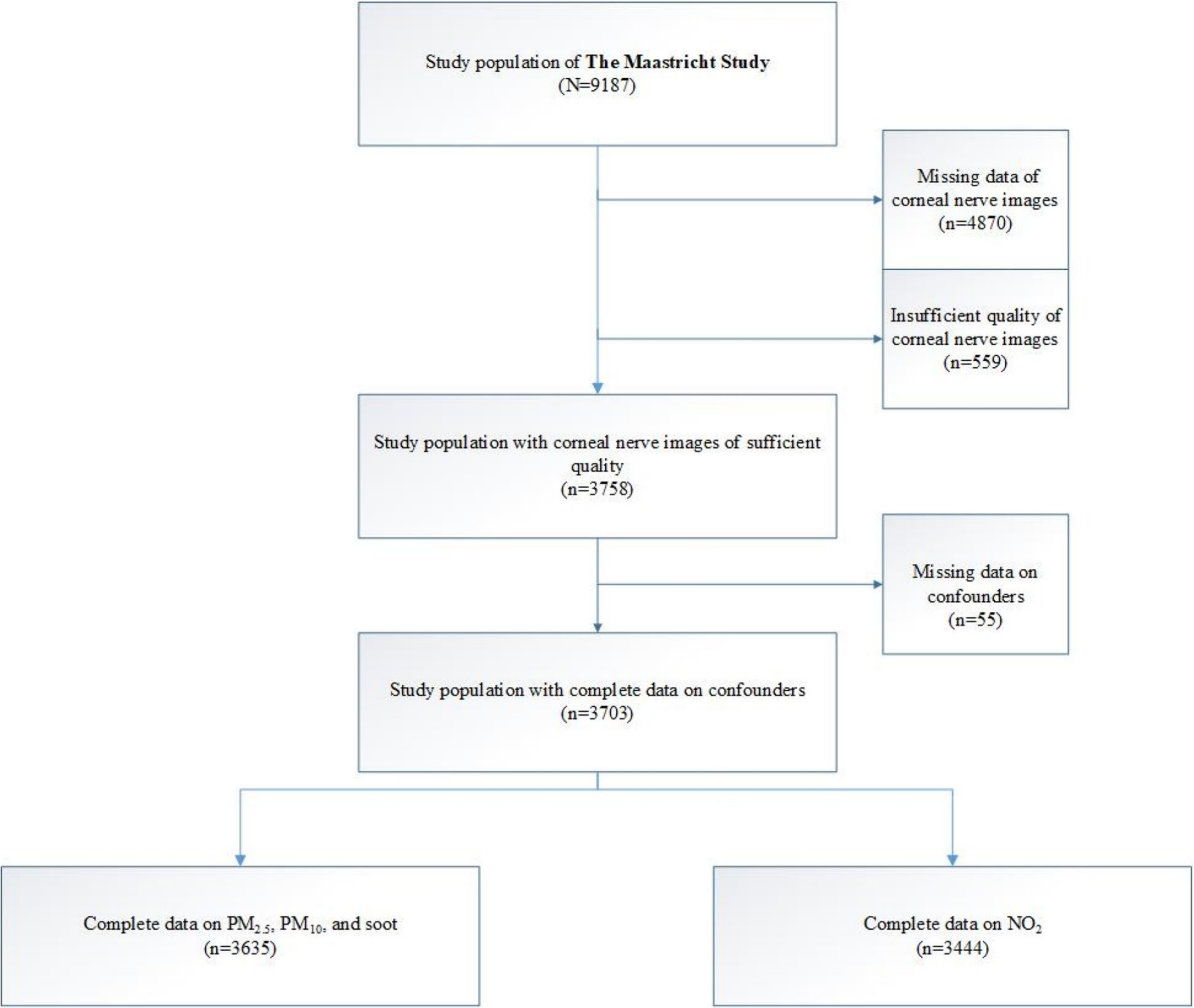
Shows the selection of participants for inclusion in the analyses

Figure 2 shows an overview of selection of the study population. Table 1 and Supplementary Table S1 show the general participant characteristics according to tertiles of composite Z-score for PM_2.5_. The study population consisted of 3635 participants with an average age of 59.3 years (± 8.7), with 51.6% being women. The population was divided into tertiles based on PM_2.5_ concentration: Tertile 1 (high), Tertile 2 (middle), and Tertile 3 (low), each comprising approximately 1200 individuals.

**Table 1:** General study population characteristics in the study population with complete data on PM_2.5_ and according to tertiles of PM_2.5_

**Characteristic**	Total study population *n* = 3635	**PM**_**2.5**_** concentration**		
		Tertile 1 (high) *n* = 1211	Tertile 2 (middle) *n* = 1219	Tertile 3 (low) *n* = 1205
Age (years)	59.3 ± 8.7	60.2 ± 8.6	59.2 ± 8.6	58.6 ± 8.8
Women	1876 (51.6)	633 (52.3)	614 (50.4)	628 (52.1)
Educational level				
High	1402 (38.6)	468 (38.6)	473 (38.8)	461 (38.3)
Medium	1017 (28.0)	308 (25.4)	344 (28.2)	365 (30.3)
Low	1216 (33.5)	435 (35.9)	402 (33.0)	379 (31.5)
Air pollutants				
PM_2.5_ (µg/m^3^)	12.2 ± 1.4	13.7 ± 0.7	12.0 ± 0.3	10.7 ± 0.6
PM_10_ (µg/m^3^)	19.0 ± 1.7	20.8 ± 0.9	18.9 ± 0.6	17.2 ± 0.7
NO_2_ (µg/m^3^)^a^	17.9 ± 3.1	17.6 ± 3.3	19.2 ± 3.0	17.0 ± 2.6
EC μg/m^3^	0.97 ± 0.1	1.0 ± 0.1	1.0 ± 0.1	0.9 ± 0.1
Corneal nerve measures				
Corneal nerve bifurcation density	73.8 ± 39.9	76.0 ± 39.1	71.4 ± 40.7	73.9 ± 39.7
Corneal nerve density	79.5 ± 24.3	80.9 ± 23.3	78.0 ± 24.9	79.6 ± 24.5
Corneal nerve length	14.9 ± 4.4	15.4 ± 4.1	14.6 ± 4.6	14.9 ± 4.5
Corneal nerve fractal dimension	1.3 ± 0.1	1.3 ± 0.1	1.3 ± 0.1	1.3 ± 0.1
Glucose metabolism status				
Normal glucose metabolism	2360 (64.9)	760 (62.8)	766 (62.8)	843 (69.2)
Prediabetes	546 (15.0)	183 (15.1)	180 (14.8)	183 (15.2)
Type 2 diabetes	722 (19.9)	267 (22.0)	269 (22.1)	186 (15.4)
Other types of diabetes	7 (0.2)	1 (0.1)	4 (0.3)	2 (0.2)
Smoking status				
Never	1401 (38.5)	462 (38.2)	463 (38.0)	476 (39.5)
Former	1787 (49.2)	618 (51.0)	595 (48.8)	574 (47.6)
Current	447 (12.3)	131 (10.8)	161 (13.2)	155 (12.9)
Alcohol consumption				
None	651 (17.9)	208 (17.2)	228 (18.7)	215 (17.8)
Low	2196 (60.4)	733 (60.5)	719 (59.0)	744 (61.7)
High	788 (21.7)	270 (22.3)	272 (22.3)	264 (20.4)

Data are presented as means ± SD or *n* (%)

^a^The sample size for total study population with no missing for NO_2_ *n* = 3444

*Abbreviations*: *SD* Standard deviation

The general characteristics of participants included in the study were found to be akin to those with missing data (see Supplementary Table S2).

**Fig. 3:**
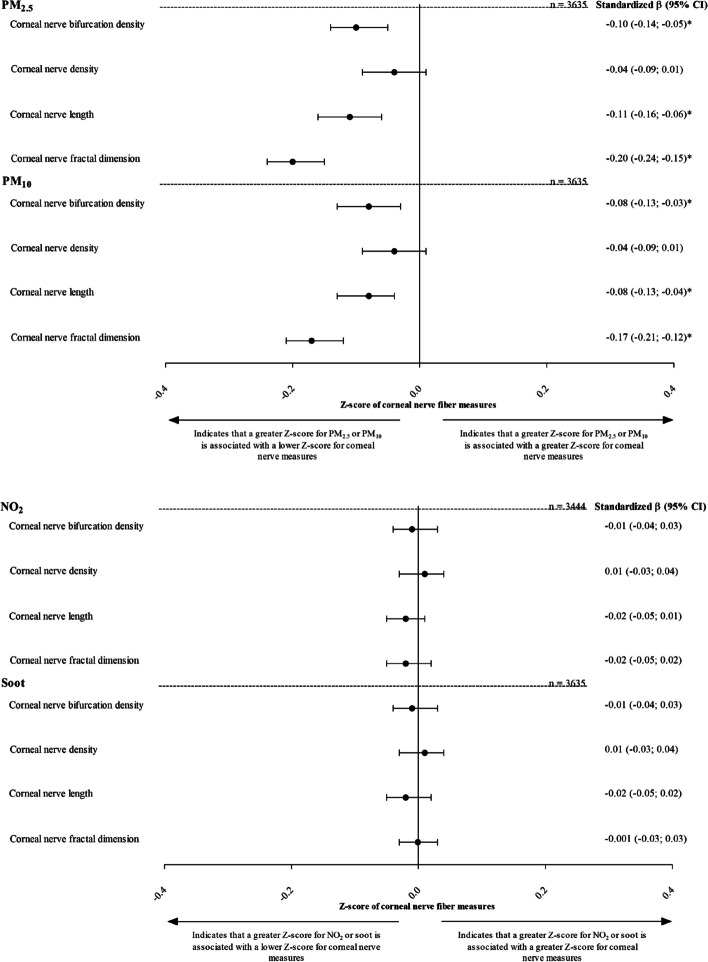
Associations of Z-score for PM_2.5_, PM_10_, NO_2_, and EC with Z-score for bifurcation density, corneal nerve density, corneal nerve length, and corneal nerve fractal dimension. Standardized regression coefficients (stβ) represent the differences in corneal nerve measures in SD per SD greater measure of air pollutants. For PM_2.5_, PM_10_, and EC, 1 SD corresponds to 39.9 number of branches/mm^2^ for corneal nerve bifurcation density, 24.3 number of main and branches/mm^2^ for corneal nerve density, 4.4 mm/mm^2^ for corneal nerve length, 0.1 (unit-less) for corneal nerve fractal dimension, 1.4 µg/m^3^ for PM_2.5_, 1.7 µg/m^3^ for PM_10_, 3.1 µg/m^3^ for NO_2_, and 0.1 µg/m3 for EC. Variables entered into model: age, sex, level of education (high, medium, low), glucose metabolism status (prediabetes and type 2 diabetes versus normal glucose metabolism), corneal confocal microscopy lag time, inclusion year of participants, smoking status (never, former, current), and alcohol consumption status (none, low, high). Asterisks indicate values that are statistically significant (*P* < 0.05). Abbreviations: CI, confidence interval; SD, standard deviation

The associations between air pollutants and corneal nerve measures are shown in Table 2 and Fig. 3. According to model 3, higher Z-scores for PM_2.5_ and PM_10_ were associated with lower Z-scores for corneal nerve bifurcation density, nerve density, nerve length, and nerve fractal dimension [stβ (95% CI): PM_2.5_ -0.10 (-0.14; -0.05), -0.04 (-0.09; 0.01), -0.11 (-0.16; -0.06), -0.20 (-0.24; -0.15); and PM_10_ -0.08 (-0.13; -0.03), -0.04 (-0.09; 0.01), -0.08 (-0.13; -0.04), -0.17 (-0.21; -0.12)], respectively.

**Table 2:** Associations of Z-scores for PM_2.5_, PM_10_, NO_2_, and EC with Z-scores for corneal nerve bifurcation density, corneal nerve density, corneal nerve length, and corneal nerve fractal dimension

Determinant	Number of participants	Model	Z-score for corneal nerve bifurcation density	Z-score for corneal nerve density	Z-score for corneal nerve length	Z-score for corneal nerve fractal dimension
			stβ (95% CI)	stβ (95% CI)	stβ (95% CI)	stβ (95% CI)
PM_2.5_	*n* = 3635	1	0.02 (-0.01; 0.05)	0.03 (-0.004; 0.06)	**0.05 (0.01; 0.08)**	**-0.08 (-0.12; -0.05)**
		2	**-0.10 (-0.15; -0.05)**	-0.04 (-0.09; 0.01)	**-0.11 (-0.16; -0.06)**	**-0.20 (-0.24; -0.15)**
		3	**-0.10 (-0.14; -0.05)**	-0.04 (-0.09; 0.01)	**-0.11 (-0.16; -0.06)**	**-0.20 (-0.24; -0.15)**
PM_10_	*n* = 3635	1	0.03 (-0.003; 0.06)	0.03 (-0.003; 0.06)	**0.06 (0.03; 0.09)**	**-0.07 (-0.10; -0.03)**
		2	**-0.08 (-0.13; -0.03)**	-0.04 (-0.09; 0.01)	**-0.09 (-0.13; -0.04)**	**-0.17 (-0.21; -0.12)**
		3	**-0.08 (-0.13; -0.03)**	-0.04 (-0.09; 0.01)	**-0.08 (-0.13; -0.04)**	**-0.17 (-0.21; -0.12)**
NO_2_	*n* = 3444	1	-0.01 (-0.04; 0.02)	0.01 (-0.03; 0.04)	-0.02 (-0.05; 0.01)	-0.02 (-0.05; 0.02)
		2	-0.01 (-0.04; 0.03)	0.01 (-0.03; 0.04)	-0.02 (-0.05; 0.01)	-0.02 (-0.05; 0.01)
		3	-0.01 (-0.04; 0.03)	0.01 (-0.03; 0.04)	-0.02 (-0.05; 0.01)	-0.02 (-0.05; 0.02)
EC	*n* = 3635	1	0.03 (-0.01; 0.06)	**0.03 (0.002; 0.07)**	**0.04 (0.003; 0.07)**	0.01 (-0.02; 0.05)
		2	-0.01 (-0.04; 0.03)	0.01 (-0.03; 0.04)	-0.02 (-0.05; 0.02)	-0.002 (-0.04; 0.03)
		3	-0.01 (-0.04; 0.03)	0.01 (-0.03; 0.04)	-0.02 (-0.05; 0.02)	-0.001 (-0.03; 0.03)

Standardized regression coefficients (stβ) represent the differences in corneal nerve measures in SD per SD greater measure of air pollutants. For PM_2.5_, PM_10_, and EC, 1 SD corresponds to 39.9 number of branches/mm^2^ for corneal nerve bifurcation density, 24.3 number of main and branches/mm^2^ for corneal nerve density, 4.4 mm/mm^2^ for corneal nerve length, 0.1 (unit-less) for corneal nerve fractal dimension, 1.4 µg/m^3^ for PM_2.5_, 1.7 µg/m^3^ for PM_10_, 3.1 µg/m^3^ for NO_2_, and 0.1 µg/m3 for EC

Bold denotes *P* < 0.05

Variables entered into models: model 1: crude; model 2: adjusted for age, sex, level of education (low, intermediate, high), glucose metabolism status (prediabetes and type 2 diabetes versus normal glucose metabolism), corneal confocal microscopy lag time, and inclusion year of participants; model 3: model 2 + smoking status (never, former, current), and alcohol consumption status (none, low, high)

*Abbreviations*: *stβ* standardized beta, *CI* Confidence interval, *SD* Standard deviation

No statistically significant associations were found between Z-scores for NO_2_ and EC with Z-scores for corneal nerve bifurcation density, nerve density, nerve length, and nerve fractal dimension [stβ (95% CI): PM_2.5_ -0.01 (-0.04; 0.03), 0.01 (-0.03; 0.04), -0.02 (-0.05; 0.01), -0.02 (-0.05; 0.02); and PM_10_ -0.01 (-0.04; 0.03), 0.01 (-0.03; 0.04), -0.02 (-0.05; 0.02), -0.001 (-0.03; 0.03)], respectively; Fig. 3 and Table 2.

Sex and glucose metabolism status did not modify the associations of Z-scores for PM_2.5_, PM_10_, NO_2_, and EC with composite Z score for corneal nerve measures [*p* value for the interaction term (potential determinant × sex; potential determinant × prediabetes versus normal glucose metabolism; potential determinant × type 2 diabetes versus normal glucose metabolism) were all < 0.05); Supplementary Table S3 and S4.

Quantitatively similar results were observed in a range of sensitivity analyses. First, associations were generally comparable to the main results after additional adjustment for dietary intake and physical activity (model 4), for ocular variables (corneal diseases, uveitis, model 5), use of glasses or contact lenses (model 6), and urbanicity (model 7) (Supplemental Table S5). Second, associations were not altered after additional adjustment for location of captured corneal nerve images (model 8), or for the orientation of the corneal nerve fibers (model 9) (Supplemental Table S6). Last, associations remained similar after replacement of level of education with occupational status (model 10) or income level (model 11) (Supplemental Table S7).

## Discussion

The primary findings of this cross-sectional observational study conducted at the population level are as follows. First, after adjustment for a range of potential confounders, higher levels of PM_2.5_ and PM_10_ were associated with lower corneal nerve measures. Second, NO_2_ and EC were not associated with corneal nerve measures. Third, the associations were not significantly different between men and women or between different glucose metabolism strata. Further, our results remained similar after additional adjustment for lifestyle factors, corneal diseases, uveitis, use of glasses or contact lenses, and urbanicity.

To the best of our knowledge, our study represents the inaugural large-scale population-based investigation unveiling novel associations between exposure to air pollutants and corneal nerve morphology. This unique association highlights a potential relationship between specific air pollutant concentrations and neurodegeneration. No earlier study has included corneal nerve measures as an outcome to investigate ocular surface disease or as a marker for neurodegeneration.

Gayraud et al. previously revealed an association between air pollutants and retinal nerve fiber layer thickness in the Alienor study population. In this study of 683 participants, they showed that higher exposure to PM_2.5_ and black carbon for 10 years was significantly associated with a faster retinal nerve fiber layer thinning during the 11-year follow-up. Moreover, no statistically significant association was found with NO_2_ [40], in line with our findings. Our findings also align with other research regarding the impact of air pollution on the ocular surface [3]. Studies have demonstrated the harmful effects of exposure to air pollutants, which may cause irritation and inflammation, resulting in conjunctivitis [3]. Air pollution may also cause tear film instability inducing the expression of inflammatory mediators in the tear film leading to dry eye disease [3]. Jurkiewicz et al. also proposed that fine particulate matter may be a risk factor for keratoconus [9].

Our results are consistent with studies demonstrating associations between air pollution exposure and neurodegeneration [41]. A systematic review and meta-analysis demonstrated a significant association between PM_2.5_ exposure and lower general cognition, verbal fluency, and executive function in individuals aged 40 and above [42]. Furthermore, Sakhvidi et al. showed that exposure to PM_2.5_ was associated with lower cognitive function in the French CONSTANCES cohort, n = 220,000 people (aged 18–69 years) [43]. Moreover in a recent longitudinal study, late life exposure to PM_2.5_ and NO_2_ was associated with Alzheimer's disease-related neurodegeneration, specifically, medial temporal lobe atrophy [44]. Indeed, recent studies have shown an association between corneal nerve loss and cognitive function in patients with mild cognitive impairment and dementia [15, 45, 46] and corneal nerve loss in subjects with mild cognitive impairment predicted the development of dementia [47]. Thus the corneal nerve loss observed could act as a surrogate marker for neurodegeneration in the brain [48].

The significant associations observed between PM_2.5_ and PM_10_ and corneal neurodegeneration, in contrast to the absence of associations with NO_2_ and EC, may be attributed to distinct mechanisms by which particulate matter affects corneal nerves. There is a strong body of data showing that different components of air pollutants, including particulate matter, carbon and nitrogen oxides have a varying impact on the nervous system and neurological disorders [49]. Longitudinal studies evaluating alterations over time are needed to provide insights into the temporal aspects of these differing associations.

As previously detailed by our research team [20] the corneal nerve metrics observed in our study population diverge from those in previous studies [50] due to several factors: (1) the assessment of corneal nerve variables in larger images, spanning an area of up to 1600 × 1600 µm, which partly encompassed the inferior whorl in certain composite images, characterized by distinct architecture, distribution, and density of corneal nerve fibers [51]; (2) the utilization of a different deep learning model (U-Net-based convolutional neural network) for fully automated tracing and analysis of corneal nerves [52]; nd (3) the definition of corneal nerve density and length differing from other studies (total number and length of corneal nerves, encompassing both main and branches, per mm2).

Our study possesses several strengths. First, to the best of our knowledge, it represents the first extensive population-based observational investigation seeking to establish the association between different air pollutants and corneal nerve morphology. Second, we adjusted for a broad range of confounders and our additional analyses generally yielded consistent findings. Third, we computed z-scores for air pollution and corneal nerve measures (continuous variables) to enable comparisons of associations between determinants and outcomes across various characteristics and units. Lastly, we revealed consistent associations with a range of different corneal nerve measures (bifurcation density, nerve density, nerve length, and nerve fractal dimension).

Our study also had certain limitations. First, due to its observational cross-sectional design, caution should be exercised in drawing any causal conclusions. However, the likelihood of reverse causation is deemed low, and we meticulously adjusted for numerous potential confounders. Secondly, despite accounting for a wide range of confounders, we cannot entirely rule out bias stemming from unmeasured confounding factors (such as workplace environment and indoor air pollution). Third, we were not able to account for change in address over time, hence we may have spuriously estimated ambient air pollution levels for those individuals who moved, potentially leading to an underestimation of associations under study. Fourth, there is a possibility that we underestimated associations due to the restricted range of distribution, as our analysis only encompassed participants residing in the Maastricht area. since corneal confocal microscopy measurements commenced in April 2013, individuals who engaged in the Maastricht Study before the initiation of corneal confocal microscopy assessment (i.e., before April 2013; n = 950; 26.1%) had a median visit interval of 5.2 years [4.9–6.1]. Although we adjusted for visit interval in our analyses to yield a more accurate estimate of the true association. Finally, this study encompassed individuals of European descent aged between 40 and 75 years residing in the Maastricht area. Further investigations are warranted to ascertain the generalizability of our findings to diverse populations and regions.

Our findings may have implications for public health policy. The significant association between exposure to PM_2.5_ and PM_10_ and corneal neurodegeneration suggests a potential need to consider enhancing air quality standards in accordance with the current European Union (EU) guidelines. Notably, our associations persist even at pollution levels below the EU's recommended thresholds, which could support discussions around revising these standards, as suggested by the WHO in 2021. In Maastricht, where our study was conducted, average concentrations of PM_2.5_ and PM_10_ (12.2 and 19.0 μg/m3, respectively) surpass WHO-recommended levels (5 and 15 μg/m3, respectively) (1) but fall below EU thresholds (25 and 40 μg/m3, respectively) (European Commission, 2008).

In conclusion, the present population-based study demonstrated that exposure to higher levels of PM_2.5_ and PM_10_ are associated with higher levels of corneal neurodegeneration, estimated from lower corneal nerve measures. Our results suggest that air pollution may be a determinant to corneal neurodegeneration and impact the ocular surface health.

## Supplementary Information

Supplementary Material 1.

## Data Availability

Data from the Maastricht Study are available to any researcher who meets the criteria for access to confidential data https://demaastrichtstudie.app/data-dictionary/. The corresponding author may be contacted to request access. Environmental exposure data can be obtained via www.gecco.nl.

## Abbreviations

PM_2.5_: Particulate Matter 2.5
PM_10_: Particulate Matter 10
NO_2_: Nitrogen Dioxide
CI: Confidence interval
SD: Standard deviation

## Data Availability

No datasets were generated or analysed during the current study.
