# Supplementary material for: Greater exposure to PM2.5 and PM10 was associated with lower corneal nerve measures: the Maastricht study - a cross-sectional study
Source: Environ Health. 2024 Sep 4;23:70. doi: 10.1186/s12940-024-01110-1 (PMC11375839; doi:10.1186/s12940-024-01110-1)
Supplement: Supplementary file 1 — Supplementary Material 1. [file 12940_2024_1110_MOESM1_ESM.docx]

Supplementary Information for

**Greater exposure to PM_2.5_ and PM_10_ was associated with lower corneal nerve measures: The Maastricht Study - a cross-sectional study**

Sara B.A. Mokhtar^1,2^; Jessica Viljoen^1^; Carla J.H. van der Kallen^1^; Tos T.J.M. Berendschot^2^; Pieter C Dagnelie, PhD^1^; Jeroen D. Albers^3,4^; Jens Soeterboek^5^; Fabio Scarpa^6^; Alessia Colonna^6^; Frank C. T. van der Heide^7^; Marleen M.J. van Greevenbroek^1^; Hans Bosm^3,4^; Abraham A. Kroon^1,8^; Rudy M.M.A. Nuijts^2^; Marlies Gijs^2^; Jeroen Lakerveld^9,10^; Rayaz A Malik^11,12^; Carroll A.B. Webers^2^; Coen D.A. Stehouwer^13^; Annemarie Koster^3,4^

^1^ Department of Internal Medicine, School for Cardiovascular Diseases, Maastricht University Medical Center, Maastricht, the Netherlands

^2^ University Eye Clinic Maastricht, School of Mental Health and Neuroscience, Maastricht University Medical Center, Maastricht, the Netherlands

^3^ Department of Social Medicine, Maastricht University, Maastricht, The Netherlands

^4^ Care and Public Health Research Institute, Maastricht University, Maastricht, The Netherlands

^5^ Alzheimer Centrum Limburg, Department of Psychiatry and Neuropsychology, School of Mental Health and Neuroscience, Maastricht University Medical Center+, Maastricht, The Netherlands

^6^ Department of Information Engineering, University of Padova, Padova, Italy

^7^ Université de Paris, Inserm U1153, Epidemiology of Ageing and Neurodegenerative diseases, Paris, France

^8^ Heart and Vascular Center, Maastricht University Medical Center, Maastricht, the Netherlands

^9^ Global Geo Health Data Center, University Medical Center Utrecht & Utrecht University, Utrecht, the Netherlands

^10^ Department of Epidemiology and Data Science, Amsterdam University Medical Centers (VUmc Location), De Boelelaan 1089a, 1081HV, Amsterdam, the Netherlands.

^11^Department of Medicine, Weill Cornell Medicine-Qatar, Qatar Foundation, Education City, Doha, Qatar

^12^Institute of Cardiovascular Science, University of Manchester, Manchester, UK

^13^Department of Chronic Diseases and Metabolism (CHROMETA), KU Leuven, Belgium

**Address for correspondence and requests for reprint**: Sara Mokhtar, MSc, Department of Internal Medicine, Department of Ophthalmology, Maastricht University Medical Center^+^, P. Debyelaan 25, P.O. Box 5800, 6202AZ Maastricht, The Netherlands, Phone: 0031-658767236 | E-mail: [s.mokhtar@maastrichtuniversity.nl](mailto:s.mokhtar@maastrichtuniversity.nl)

### **Supplemental Material**

**Content**

Supplemental Methods

Supplemental Figures

Supplemental Tables

**Content**

- **Supplemental Table S1** Additional general study population characteristics according to tertiles of PM_2.5_ concentrations in the study population with complete data on PM_2.5_
- **Supplemental Table S2** General study population characteristics of the included and excluded participants
- **Supplemental Table S3** P-values for the interaction term of sex with determinants under the study in the associations of Z-scores for PM_2.5_, PM_10_, NO_2_, and EC with Z-scores for corneal nerve bifurcation density, nerve density, nerve length, and fractal dimension
- **Supplemental Table S4** P-values for the interaction term of glucose metabolism status with determinants under the study in the associations of Z-scores for PM_2.5_, PM_10_, NO_2_, and EC with Z-scores for corneal nerve bifurcation density, nerve density, nerve length, and fractal dimension
- **Supplemental Table S5** Associations of Z-scores for PM_2.5_, PM_10_, NO_2_, and EC with Z-scores for corneal nerve bifurcation density, corneal nerve density, corneal nerve length, and corneal nerve fractal dimension additionally adjusted for life style factors (dietary intake (except for alcohol) and physical activity, model 4), for ocular variables (corneal diseases, uveitis, model 5), use of glasses or contact lenses (model 6), and urbanicity (model 7)
- **Supplemental Table S6** Associations of Z-scores for PM_2.5_, PM_10_, NO_2_, and EC with Z-scores for corneal nerve bifurcation density, corneal nerve density, corneal nerve length, and corneal nerve fractal dimension additionally adjusted for: location of captured corneal nerve images (model 8), the orientation of the corneal nerve fibres (model 9)
- **Supplemental Table S7** Associations of Z-scores for PM_2.5_, PM_10_, NO_2_, and EC with Z-scores for corneal nerve bifurcation density, corneal nerve density, corneal nerve length, and corneal nerve fractal dimension after replacement of educational level with occupational status (model 10) or income level (model 11)

**Supplemental Methods**

**Assessment of corneal confocal microscopy measurements**

Individuals who had a corneal transplant or had a corneal infection of the left eye were excluded from the measurements. Participants were instructed to fix their vision on a white light throughout the scan. Trained research assistants imaged the sub-basal nerve plexus in the central part of the cornea according to a standard operating procedure. We assessed multiple images (400×400 μm; 384×384 pixels, 8-bit) assembled using a composite algorithm implemented in the HRT3 user interface (Heidelberg Engineering), as previously described [1]. Real-time mapping was performed on an area up to 1600×1600 µm (1536×1536 pixels, 8-bit) that partially included the inferior whorl in some of the composite images. No measures were taken to include or exclude this region. The advantage of performing large scale corneal confocal microscopy imaging (1600 × 1600 μm) is described previously

**Quality assessment protocol for corneal erve measures**

According to the selection criteria, images were considered to be usable (*n*=3758) if 50% or more of the total captured area was of good quality. For example, a fully captured area consists of 16 sub-squares. If at least 8 sub-squares were of good quality, the image was included. For assessment, we scored the segmented images (number of good quality sub-images) based on the contrast, the depth, the sharpness, the focus position, and the presence of pressure lines (Supplemental Figure S2). We also assessed the location of the captured images based on the orientation of the corneal nerves (i.e., in the center of the cornea, corneal nerves are thought to be vertical, peripherally, corneal nerves are thought to be more horizontal, and the Inferior whorl, corneal nerves are thought to show a swirl pattern, (Supplemental Figure S3). The intra-class and inter-observer correlation coefficients, both indices of reliability, were ≥ 0.97 and ≥ 0.89, respectively [2]. The percentage of agreement of quality image assessment between observers was on average 97.2%.

**Supplemental Figures**


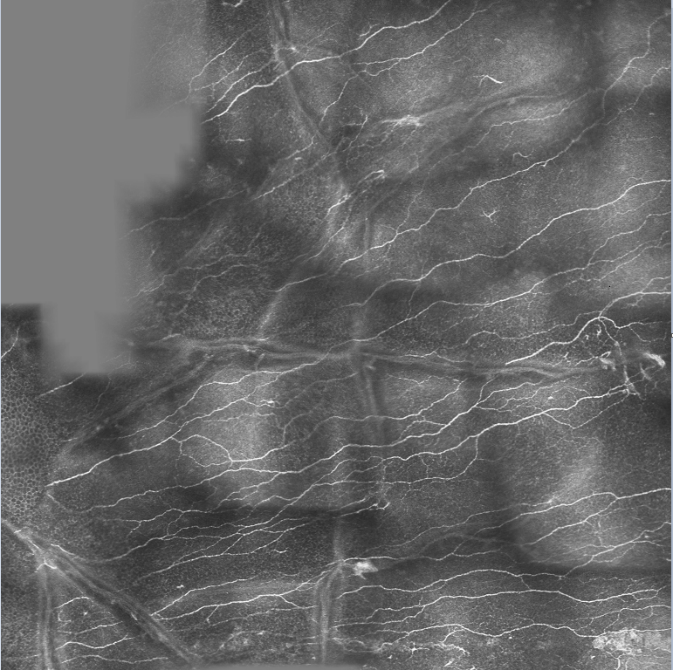

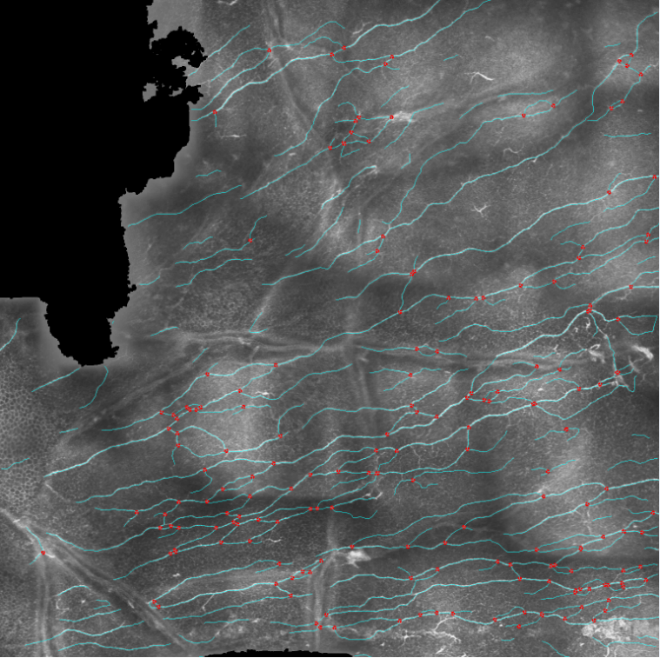


**(a) (b)**

**Supplemental Figure S1.** (a) Original CCM image (b) Automatically detected nerves


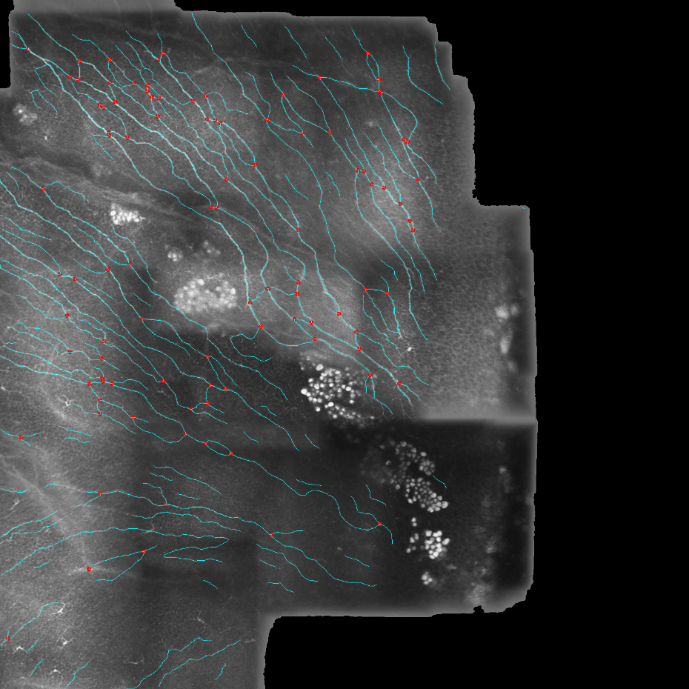

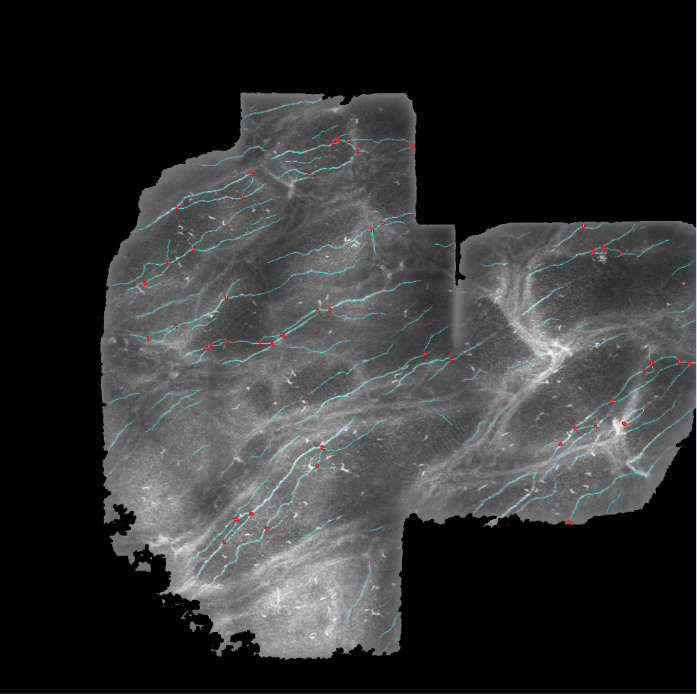


**(a) (b)**

**Supplemental Figure S2.** Example of (a) Included image (b) Excluded image


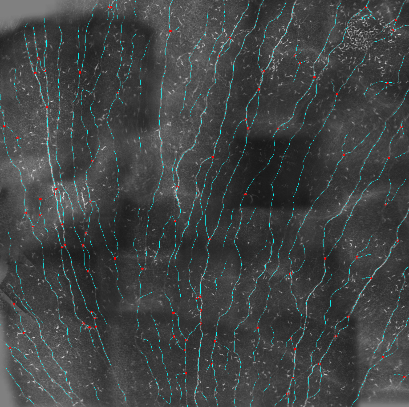

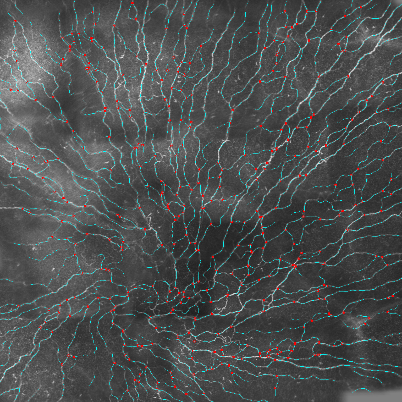


**(a) (b)**


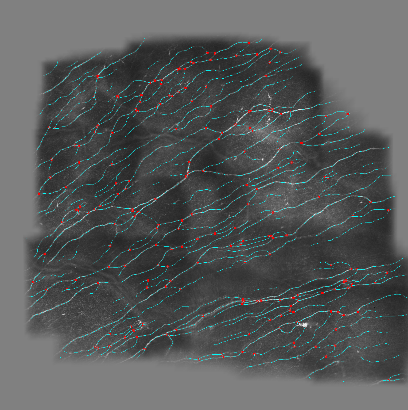

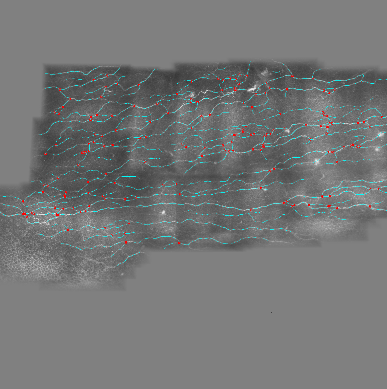


**(c) (d)**

**Supplemental Figure S3. (a) Center of the cornea (b) Inferior nasal (c and d) Peripheral**

**Supplemental Tables**

**Supplemental Table S1.** Additional general study population characteristics according to tertiles of PM_2.5_ concentrations in the study population with complete data on PM_2.5_

|  |  |  | **PM2.5 concentration** | | |  |
| --- | --- | --- | --- | --- | --- | --- |
| Characteristic | Number of participants | Total study  population | Tertile 1  (high)  n= 1211 | Tertile 2  (middle)  n= 1219 | Tertile 3  (low)  n= 1205 | Number of participants with missing data |
| Dutch Healthy diet score (points) | 3381 | 77.0 ± 14.6 | 77.6 ± 14.5 | 76.4 ± 14.9 | 76.4 ± 14.9 | 254 |
| Physical activity (hours/day) | 3250 | 2.0 ± 0.7 | 2.0 ± 0.7 | 1.9 ± 0.7 | 1.9 ± 0.7 | 385 |
| Income (euro) | 2843 | 1875.0 [1502.6 – 2525.9] | 1856.15 [1502.6 – 2386.5] | 1875.0 [1502.6 – 2525.9] | 1956.6 [1502.6 – 2563.3] | 792 |
| Ocular disorder, yes vs. no |  |  |  |  |  |  |
| Corneal diseases | 3340 | 45 (1.3) | 11 (1.0) | 13 (1.2) | 21 (1.7) | 295 |
| Uveitis | 3341 | 86 (2.6) | 31 (2.7) | 28 (2.5) | 27 (2.5) | 264 |
| Use of glasses | 3423 | 2219 (64.8) | 729 (65.3) | 771 (65.5) | 719 (63.7) | 212 |
| Use of contact lenses | 3434 | 347 (10.1) | 107 (8.8) | 117 (9.6) | 123 (10.8) | 201 |
| Corneal confocal microscopy scans not performed at baseline | 950 | 950 (26.1) | 33 (2.7) | 525 (43.1) | 392 (32.5) | / |
| Corneal confocal microscopy lag time (years) | 950 | 5.2 [4.9-5.8] | 5.8 [5.0-6.1] | 5.3 [5.0-5.8] | 5.1 [4.6-5.3] | / |

Data are presented as means ± SD, median [IQR] or *n* (%)

**Supplemental Table S2.** General study population characteristics of the included and excluded participants

| **Characteristic** | **Included study  population** (n = 3635) | **Missing data in/excluded** | **Excluded study population**  (n =5552) |
| --- | --- | --- | --- |
| Age (years) | 59.3 ± 8.7 | 0/0 | 59.7 ± 8.8 |
| Women | 1876 (51.6) | 0/0 | 2741 (49.4) |
| Educational level |  | 0/128 |  |
| High | 1402 (38.6) |  | 2155 (39.7) |
| Intermediate | 1017 (28.0) |  | 1481 (27.3) |
| Low | 1216 (33.5) |  | 1788 (33.0) |
| Air pollutants* |  |  |  |
| PM_2.5_ | 12.2 ± 1.4 | 0/58 | 12.2 ± 1.5 |
| PM_10_ | 19.0 ± 1.7 | 0/58 | 19.0 ± 1.8 |
| NO_2_ | 17.9 ± 3.1 | 191/55 | 18.3 ± 3.2 |
| EC | 0.97 ± 0.1 | 0/58 | 0.97 ± 0.13 |
| Corneal nerve measures** |  | 0/55 |  |
| Corneal nerve bifurcation density | 73.8 ± 39.9 |  | 75.8 ± 60.3 |
| Corneal nerve density | 79.5 ± 24.3 |  | 80.4 ± 33.4 |
| Corneal nerve length | 14.9 ± 4.4 |  | 14.7 ± 4.7 |
| Corneal nerve fractal dimension | 1.3 ± 0.1 |  | 1.3 ± 0.1 |
| Glucose metabolism status |  | 0/0 |  |
| Normal glucose metabolism | 2360 (64.9) |  | 3387 (61.0) |
| Prediabetes | 546 (15.0) |  | 836 (15.1) |
| Type 2 diabetes | 722 (19.9) |  | 1282 (23.1) |
| Other types of diabetes | 7 (0.2) |  | 47 (0.8) |
| Smoking status |  | 0/73 |  |
| Never | 1401 (38.5) |  | 2101 (38.3) |
| Former | 1787 (49.2) |  | 2624 (47.9) |
| Current | 447 (12.3) |  | 754 (13.8) |
| Alcohol consumption |  | 0/75 |  |
| None | 651 (17.9) |  | 1041 (19.0) |
| Low | 2196 (60.4) |  | 3188 (58.2) |
| High | 788 (21.7) |  | 1248 (22.8) |

Data are presented as means ± SD, median [IQR] or *n* (%)

^*^ Number of participants with missing data represents the number of participants that had known data on air pollutant concentration and were excluded due to missing confounders

^**^ Number of participants with missing data represents the number of participants that had corneal nerve images of sufficient quality and were excluded due to missing confounders

**Supplemental Table S3.** P-values for the interaction term of sex with determinants under the study in the associations of Z-scores for PM_2.5_, PM_10_, NO_2_, and EC with Z-scores for corneal nerve bifurcation density, nerve density, nerve length, and fractal dimension

| Determinant | Number of participants | Z-score of corneal nerve bifriction density | Z-score of corneal nerve fiber density | Z-score of corneal nerve fiber density | Z-score of corneal nerve fiber density |
| --- | --- | --- | --- | --- | --- |
|  |  | P-value (Sex × determinant) | | | |
| PM_2.5_ | 3635 | 0.33 | 0.37 | 0.26 | 0.59 |
| PM_10_ | 3635 | 0.36 | 0.44 | 0.30 | 0.73 |
| NO_2_ | 3444 | 0.51 | 0.56 | 0.65 | 0.71 |
| EC | 3635 | 0.24 | 0.22 | 0.51 | 0.75 |

*P*-values represent the *P*-values for the interaction terms of sex with determinant under the study (e.g., sex × PM_2.5_) in the associations of air pollutants with corneal nerve measures. Variables in the model in addition to determinants and interaction term(s) with sex are: age, sex, educational level , glucose metabolism status (prediabetes and type 2 diabetes versus normal glucose metabolism), corneal confocal microscopy visit interval, inclusion year of participants, smoking status (never, former, current), and alcohol consumption status (none, middle, high). In addition, we added an interaction term for all confounders (e.g., age × sex, systolic blood pressure × sex)

**Supplemental Table S4.** P-values for the interaction term of glucose metabolism status with determinants under the study in the associations of Z-scores for PM_2.5_, PM_10_, NO_2_, and EC with Z-scores for corneal nerve bifurcation density, nerve density, nerve length, and fractal dimension

| Determinant | Number of participants | Z-score of corneal nerve bifriction density | | Z-score of corneal nerve fiber density | | Z-score of corneal nerve fiber density | Z-score of corneal nerve fiber density | |
| --- | --- | --- | --- | --- | --- | --- | --- | --- |
|  |  | **P-value (Prediabetes vs normal glucose metabolism × determinant)** | | | | | | |
| PM_2.5_ | 3635 | 0.31 | | 0.24 | | 0.15 | 0.13 | |
| PM_10_ | 3635 | 0.47 | | 0.37 | | 0.19 | 0.12 | |
| NO_2_ | 3444 | 0.85 | | 0.83 | | 0.59 | 0.54 | |
| EC | 3635 | 0.90 | | 0.81 | | 0.46 | 0.49 | |
|  |  | **P-value (type 2 diabetes vs normal glucose metabolism × determinant)** | | | | | | |
| PM_2.5_ | 3635 | 0.63 | 0.98 | | 0.69 | | | 0.20 |
| PM_10_ | 3635 | 0.87 | 0.66 | | 0.94 | | | 0.60 |
| NO_2_ | 3444 | 0.53 | 0.80 | | 0.33 | | | 0.35 |
| EC | 3635 | 0.45 | 0.50 | | 0.38 | | | 0.76 |

*p*-values represent the *p*-values for the interaction terms of glucose metabolism status (prediabetes and type 2 diabetes versus normal glucose metabolism) with determinant under the study (e.g., prediabetes vs normal glucose metabolism × PM_2.5_ or type 2 diabetes vs normal glucose metabolism × PM_2.5_ ) in the associations of air pollutants with corneal nerve measures. Variables in the model in addition to determinants and interaction term(s) with prediabetes and type 2 diabetes versus normal glucose metabolism are: age, sex, educational level, corneal confocal microscopy visit interval, inclusion year of participants, office systolic blood pressure, total cholesterol-to-HDL cholesterol ratio, use of antihypertensive and/ or lipid-modifying medication, waist circumference smoking status (never, former, current), and alcohol consumption status (none, middle, high). In addition, we added an interaction terms for all confounders (e.g., age × prediabetes vs normal glucose metabolism, systolic blood pressure × type 2 diabetes vs normal glucose metabolism)

**Supplemental Table S5.** Associations of Z-scores for PM_2.5_, PM_10_, NO_2_, and EC with Z-scores for corneal nerve bifurcation density, corneal nerve density, corneal nerve length, and corneal nerve fractal dimension additionally adjusted for life style factors (dietary intake (except for alcohol) and physical activity, model 4), for ocular variables (corneal diseases, uveitis, model 5), use of glasses or contact lenses (model 6), and urbanicity (model 7)

| Determinant | Z-score for corneal nerve bifurcation density  stβ (95% CI) | Z-score for corneal nerve density  stβ (95% CI) | Z-score for corneal nerve length  stβ (95% CI) | Z-score for corneal nerve fractal dimension  stβ (95% CI) |
| --- | --- | --- | --- | --- |
|  | **Model 4**  **N= (3030)** | **Model 4**  **N= (3030)** | **Model 4**  **N= (3030)** | **Model 4**  **N= (3030)** |
| PM_2.5_ | **-0.09 (-0.14; -0.04)** | -0.03 (-0.09; 0.02) | **-0.10 (-0.16; -0.05)** | **-0.21 (-0.26; -0.15)** |
| PM_10_ | **-0.07 (-0.12; -0.02)** | -0.03 (-0.09; 0.02) | **-0.08 (-0.13; -0.02)** | **-0.17 (-0.23; -0.12)** |
| NO_2_* | -0.004 (-0.04; 0.03) | 0.01 (-0.03; 0.05) | -0.02 (-0.05; 0.02) | -0.02 (-0.05; 0.02) |
| EC | 0.003 (-0.03; 0.04) | 0.02 (-0.02; 0.05) | -0.004 (-0.04; 0.03) | 0.01 (-0.03; 0.05) |
|  | **Model 5**  **N= (3199)** | **Model 5**  **N= (3199)** | **Model 5**  **N= (3030)** | **Model 5**  **N= (3199)** |
| PM_2.5_ | **-0.09 (-0.14; -0.04)** | -0.03 (-0.08; 0.02) | **-0.10 (-0.15; -0.05)** | **-0.19 (-0.25; -0.14)** |
| PM_10_ | **-0.07 (-0.12; -0.02)** | -0.03 (-0.08; 0.02) | **-0.08 (-0.13; -0.02)** | **-0.16 (-0.22; -0.11)** |
| NO_2_* | -0.000 (-0.04; 0.04) | 0.01 (-0.02; 0.05) | -0.01 (-0.05; 0.02) | -0.01 (-0.05; 0.02) |
| EC | -0.03 (-0.04; 0.03) | 0.01 (-0.03; 0.05) | -0.01 (-0.05; 0.02) | 0.000 (-0.04; 0.04) |
|  | **Model 6**  **N= (3407)** | **Model 6**  **N= (3407)** | **Model 6**  **N= (3407)** | **Model 6**  **N= (3407)** |
| PM_2.5_ | **-0.09 (-0.14; -0.03)** | -0.03 (-0.08; 0.02) | **-0.10 (-0.15; -0.05)** | **-0.16 (-0.21; -0.11)** |
| PM_10_ | **-0.07 (-0.12; -0.01)** | -0.03 (-0.08; 0.03) | **-0.07 (-0.13; -0.02)** | **-0.13 (-0.18; -0.08)** |
| NO_2_* | -0.01 (-0.04; 0.03) | 0.01 (-0.03; 0.04) | -0.02 (-0.06; 0.01) | -0.02 (-0.06; 0.01) |
| EC | -0.01 (-0.05; 0.03) | 0.01 (-0.03; 0.04) | -0.02 (-0.06; 0.01) | -0.01 (-0.05; 0.02) |
|  | **Model 7**  **N= (3624)** | **Model 7**  **N= (3624)** | **Model 7**  **N= (3624)** | **Model 7**  **N= (3624)** |
| PM_2.5_ | **-0.09 (-0.14; -0.05)** | -0.04 (-0.09; 0.01) | **-0.11 (-0.16; -0.06)** | **-0.19 (-0.24; -0.15)** |
| PM_10_ | **-0.07 (-0.13; -0.03)** | -0.04 (-0.09; 0.01) | **-0.08 (-0.13; -0.04)** | **-0.16 (-0.21; -0.12)** |
| NO_2_* | -0.01 (-0.04; 0.03) | 0.01 (-0.03; 0.04) | -0.02 (-0.05; 0.02) | -0.02 (-0.05; 0.02) |
| EC | -0.01 (-0.04; 0.03) | 0.01 (-0.03; 0.04) | -0.02 (-0.05; 0.02) | 0.000 (-0.03; 0.03) |

Standardized regression coefficients (stβ) represent the differences in corneal nerve fiber measures in SD per SD greater measure of air pollutants. For PM_2.5_, PM_10_, and EC, 1 SD corresponds to 39.6 number of branches/mm^2^ for corneal nerve bifurcation density, 24.2 number of main and branches/mm^2^ for corneal nerve density, 4.4 mm/mm^2^ for corneal nerve length, 0.1 (unit-less) for corneal nerve fractal dimension, 1.4 µg/m^3^ for PM_2.5_, 1.7 µg/m^3^ for PM_10_, 3.1 µg/m^3^ for NO_2_, and 0.1 µg/m3 for EC. Models 4, 5, 6 and 7 values per SD were numerically comparable

Bold denotes P<0.05

Variables entered into the models: age, sex, educational level (low, intermediate, high), glucose metabolism status (prediabetes and type 2 diabetes versus normal glucose metabolism), corneal confocal microscopy lag time, inclusion year of participants, smoking status (never, former, current), and alcohol consumption status (none, low, high).

^*^ Number of participants with known data on NO_2_ for model 4 n=2859; for model 5 n=3027; for model 6 n=3289; for model 7 n=3433.

Abbreviations: stβ, standardized beta; CI, confidence interval; SD, standard deviation

**Supplemental Table S6.** Associations of Z-scores for PM_2.5_, PM_10_, NO_2_, and EC with Z-scores for corneal nerve bifurcation density, corneal nerve density, corneal nerve length, and corneal nerve fractal dimension additionally adjusted for location of captured corneal nerve images (model 8), and for the orientation of the corneal nerve fibres (model 9)

| Determinant | Z-score for corneal nerve bifurcation density  stβ (95% CI) | Z-score for corneal nerve density  stβ (95% CI) | Z-score for corneal nerve length  stβ (95% CI) | Z-score for corneal nerve fractal dimension  stβ (95% CI) |
| --- | --- | --- | --- | --- |
|  | **Model 8**  **N= (3631)** | **Model 8**  **N= (3631)** | **Model 8**  **N= (3631)** | **Model 8**  **N= (3631)** |
| PM_2.5_ | **-0.09 (-0.14; -0.04)** | -0.05 (-0.09; 0.002) | **-0.10 (-0.14; -0.05)** | **-0.16 (-0.21; -0.11)** |
| PM_10_ | **-0.07 (-0.12; -0.02)** | -0.04 (-0.09; 0.01) | **-0.07 (-0.12; -0.02)** | **-0.13 (-0.18; -0.09)** |
| NO_2_* | -0.01 (-0.05; 0.02) | 0.002 (-0.03; 0.03) | -0.02 (-0.06; 0.01) | -0.03 (-0.06; 0.01) |
| EC | -0.01 (-0.05; 0.02) | 0.003 (-0.03; 0.04) | -0.02 (-0.05; 0.01) | -0.01 (-0.04; 0.02) |
|  | **Model 9**  **N= (3515)** | **Model 9**  **N= (3515)** | **Model 9**  **N= (3515)** | **Model 9**  **N= (3515)** |
| PM_2.5_ | **-0.09 (-0.14; -0.04)** | -0.03 (-0.08; 0.02) | **-0.11 (-0.16; -0.06)** | **-0.21 (-0.26; -0.16)** |
| PM_10_ | **-0.07 (-0.12; -0.02)** | -0.03 (-0.08; 0.02) | **-0.08 (-0.13; -0.04)** | **-0.17 (-0.22; -0.12)** |
| NO_2_* | -0.004 (-0.04; 0.03) | 0.01 (-0.02; 0.04) | -0.02 (-0.05; 0.02) | -0.02 (-0.05; 0.02) |
| EC | -0.002 (-0.04; 0.03) | 0.01 (-0.02; 0.05) | -0.01 (-0.05; 0.02) | 0.000 (-0.03; 0.03) |

Standardized regression coefficients (stβ) represent the differences in corneal nerve fiber measures in SD per SD greater measure of air pollutants. For PM_2.5_, PM_10_, and EC, 1 SD corresponds to 39.6 number of branches/mm^2^ for corneal nerve bifurcation density, 24.2 number of main and branches/mm^2^ for corneal nerve density, 4.4 mm/mm^2^ for corneal nerve length, 0.1 (unit-less) for corneal nerve fractal dimension, 1.4 µg/m^3^ for PM_2.5_, 1.7 µg/m^3^ for PM_10_, 3.1 µg/m^3^ for NO_2_, and 0.1 µg/m3 for EC. Models 8 and 9 values per SD were numerically comparable

Bold denotes P<0.05

Variables entered into the models: age, sex, educational level (low, intermediate, high), glucose metabolism status (prediabetes and type 2 diabetes versus normal glucose metabolism), corneal confocal microscopy lag time, inclusion year of participants, smoking status (never, former, current), and alcohol consumption status (none, low, high).

^*^ Number of participants with known data on NO_2_ for model 8 n=3440; for model 9 n=3324

Abbreviations: stβ, standardized beta; CI, confidence interval; SD, standard deviation

**Supplemental Table S7.** Associations of Z-scores for PM_2.5_, PM_10_, NO_2_, and EC with Z-scores for corneal nerve bifurcation density, corneal nerve density, corneal nerve length, and corneal nerve fractal dimension after replacement of educational level with occupational status (model 10) or income level (model 11)

| Determinant | Z-score for corneal nerve bifurcation density  stβ (95% CI) | Z-score for corneal nerve density  stβ (95% CI) | Z-score for corneal nerve length  stβ (95% CI) | Z-score for corneal nerve fractal dimension  stβ (95% CI) |
| --- | --- | --- | --- | --- |
|  | **Model 10**  **N= (3280)** | **Model 10**  **N= (3280)** | **Model 10**  **N= (3280)** | **Model 10**  **N= (3280)** |
| PM_2.5_ | **-0.10 (-0.15; -0.05)** | -0.05 (-0.10; 0.01) | **-0.12 (-0.17; -0.07)** | **-0.20 (-0.25; -0.15)** |
| PM_10_ | **-0.09 (-0.14; -0.04)** | -0.05 (-0.10; 0.004) | **-0.09 (-0.14; -0.04)** | **-0.17 (-0.00; -0.12)** |
| NO_2_* | -0.01 (-0.05; 0.02) | 0.004 (-0.03; 0.04) | -0.02 (-0.05; 0.02) | -0.02 (-0.05; 0.02) |
| EC | -0.01 (-0.05; 0.02) | 0.003 (-0.03; 0.04) | -0.02 (-0.05; 0.02) | -0.003 (-0.04; 0.03) |
|  | **Model 11**  **N= (2843)** | **Model 11**  **N= (2843)** | **Model 11**  **N= (2843)** | **Model 11**  **N= (2843)** |
| PM_2.5_ | **-0.10 (-0.15; 0.04)** | -0.03 (-0.09; 0.02) | **-0.10 (-0.15; -0.05)** | **-0.19 (-0.25; -0.14)** |
| PM_10_ | **-0.07 (-0.13; -0.02)** | -0.03 (-0.08; 0.03) | **-0.07 (-0.12; -0.01)** | **-0.16 (-0.22; -0.11)** |
| NO_2_* | -0.004 (-0.04; 0.03) | 0.02 (-0.02; 0.05) | -0.01 (-0.04; 0.03) | -0.01 (-0.05; 0.03) |
| EC | -0.01 (-0.05; 0.03) | 0.01 (-0.02; 0.05) | -0.01 (-0.05; 0.03) | -0.01 (-0.04; 0.03) |

Standardized regression coefficients (stβ) represent the differences in corneal nerve fiber measures in SD per SD greater measure of air pollutants. For PM_2.5_, PM_10_, and EC, 1 SD corresponds to 39.7 number of branches/mm^2^ for corneal nerve bifurcation density, 24.3 number of main and branches/mm^2^ for corneal nerve density, 4.4 mm/mm^2^ for corneal nerve length, 0.1 (unit-less) for corneal nerve fractal dimension, 1.4 µg/m^3^ for PM_2.5_, 1.7 µg/m^3^ for PM_10_, 3.1 µg/m^3^ for NO_2_, and 0.1 µg/m3 for EC. Models 10 and 11 values per SD were numerically comparable

Bold denotes P<0.05

Variables entered into the models: age, sex, glucose metabolism status (prediabetes and type 2 diabetes versus normal glucose metabolism), corneal confocal microscopy lag time, inclusion year of participants, smoking status (never, former, current), and alcohol consumption status (none, low, high).

^*^ Number of participants with known data on NO_2_ for model 10 n=3109; for model 11 n=2689

Abbreviations: stβ, standardized beta; CI, confidence interval; SD, standard deviation

**References**

[1] Mokhtar SBA, van der Heide FCT, Oyaert KAM, et al. (2023) (Pre)diabetes and a higher level of glycaemic measures are continuously associated with corneal neurodegeneration assessed by corneal confocal microscopy: the Maastricht Study. Diabetologia 66(11): 2030-2041. 10.1007/s00125-023-05986-5

[2] De Clerck EEB, Schouten J, Berendschot T, et al. (2020) Reduced corneal nerve fibre length in prediabetes and type 2 diabetes: The Maastricht Study. Acta Ophthalmol 98(5): 485-491. 10.1111/aos.14359
